# Axillary and rectal thermometry in the newborn: do they agree?

**DOI:** 10.1186/1756-0500-7-584

**Published:** 2014-08-31

**Authors:** Lama Charafeddine, Hani Tamim, Habiba Hassouna, Randa Akel, Mona Nabulsi

**Affiliations:** Department of Pediatrics and Adolescent Medicine, American University of Beirut Medical Center, P.O. Box: 11–0236, Riad El-Solh, 1107 2020 Beirut, Lebanon; Biostatistics Unit, Clinical Research Institute, Faculty of Medicine, American University of Beirut, Beirut, Lebanon; Department of Internal Medicine, American University of Beirut, Beirut, Lebanon; Department of Dermatology, American University of Beirut, Beirut, Lebanon

**Keywords:** Axillary temperature, Diagnostic accuracy, Newborn, Rectal temperature, Thermometry methods

## Abstract

**Background:**

Accurate measurement of body temperature is critical for the assessment of a newborn’s general well-being. In nursery settings, the gold standard rectal thermometry has been replaced by the axillary method. However, evidence pertaining to the agreement between axillary and rectal thermometry in the newborn is controversial. In this cross-sectional study, the agreement between axillary and rectal temperature in newborns, as well as the effects of neonatal, maternal and environmental factors on this agreement were investigated.

**Methods:**

The *mean difference* between axillary and rectal temperatures was compared in stable term and preterm newborns using paired t-test for the means of differences, Pearson correlation coefficient (*r*), and the Bland-Altman plot. Stepwise multivariate regression assessed predictors of this difference in the overall group and by gestational age categories.

**Results:**

The study included 118 newborns with gestational ages ranging from 29 to 41 weeks, median birth weight of 2980 grams (IQR: 2321.3-3363.8). Axillary and rectal temperatures correlated significantly (*r* = 0.5, p = 0.000) and had similar overall means but differed in 34–36 weeks gestation newborns (p = 0.01). Correlation between both methods increased with advancing gestational age being highest in term newborns (*r* = 0.6, p = 0.000). Bland-Altman plots revealed good agreement in gestational ages above 29 weeks. The difference between measurements increased with Cesarean delivery (*ß* = 0.2; 95% CI: 0.02, 0.38), but decreased with advancing chronological age (*ß* = -0.01; 95% CI: -0.02,-0.01), and with gestational age (*ß* = -0.05; 95% CI: -0.08,-0.01).

**Conclusion:**

In clinically stable term and preterm infants, axillary thermometry is as reliable as rectal measurement. Predictors of agreement between the two methods include gestational age, chronological age and mode of delivery. Further studies are needed to confirm this agreement in sick newborns and in extremely premature infants.

## Background

Body temperature is an essential vital sign that reflects the wellbeing of a newborn. Temperature variation can be an indication of maladaptation to the external environment, as well as a sign of serious illness. Hence, accurate measurement of a newborn’s body temperature is critical for early detection of serious conditions, and for appropriate and timely intervention or treatment. Rectal thermometry, the gold standard method of temperature measurement is more invasive than skin or tympanic thermometry [1], and has therefore been replaced by the less invasive axillary method in nursery settings, including neonatal intensive care units (NICU) [1–3]. However, evidence pertaining to the agreement between axillary and rectal temperature measurements in the newborn is controversial, with conflicting results regarding the accuracy and precision of axillary temperature.

A systematic search of the literature for studies comparing axillary and rectal thermometry in the newborn reveals one systematic review [4] that identified two studies in neonates with opposite results and significant heterogeneity. Subsequently, seven original studies were published revealing controversial results [3, 5–10]. In four studies [5–7, 9] there was poor agreement between rectal and axillary measurements using the Bland-Altman method [11], whereas two studies reported good correlation between axillary and rectal temperature measurements [3, 10]. In the seventh study [8], skin temperature measured from the back correlated with rectal measurement better than skin temperature obtained from the abdomen. The level of agreement between the two methods was reported only by Friedrichs, et al. [10].

In view of the existing controversy, we conducted this study that aims at assessing the agreement between axillary and rectal thermometry in term and preterm neonates of different gestational ages, as well as identifying the neonatal, maternal or environmental factors that may affect this agreement.

## Methods

This was an observational, cross-sectional study conducted in the Normal Nursery and Neonatal Intensive Care Unit (NICU) of the American University of Beirut Medical Center (AUBMC), Lebanon. Between December 2012 and July 2013, all newborns who were admitted to the Normal Nursery or NICU were screened for inclusion in the study. Neonates whose age was less than six hours were excluded, as well as those who suffered from any of the following conditions: critical clinical status, necrotizing enterocolitis, disseminated intravascular coagulation, bleeding disorders or thrombocytopenia, immunodeficiency, intraventricular hemorrhage, congenital anomalies, therapeutic-induced hypothermia, neurologic disorders, and rectal pathology such as rectal injury, imperforate anus, or rectal surgery.

Neonates satisfying the inclusion criteria were subjected to axillary and rectal temperature measurements after obtaining parental written informed consent. For each neonate, one paired temperature recording was performed in the same sequence by the same investigator (MN): one axillary temperature reading (less invasive method) followed immediately by one rectal temperature reading (more invasive method), using the same digital thermometer Welch Allyn® Sure Temp® Plus Model 690 (Welch Allyn, Inc., San Diego, California), according to the manufacturer’s instructions for proper device use. Rectal measurements were obtained by gentle insertion of the rectal probe two centimeters into the rectum.

For each neonate the following data were collected: gestational age, chronological age, gender, birth weight, birth length, head circumference, mode of delivery, mode of maternal anesthesia, resuscitation at delivery and type of resuscitation; admission status and type of placement (crib, normal humidity isolette, high humidity isolette, warmer). For newborns who were admitted to NICU, we also recorded the *Newborn’s Clinical Risk Index for Babies* (CRIB) Score [12] along with the initial and current diagnosis.

### Statistical analyses

Neonates were divided into four categories according to their gestational age: term (≥37 weeks), late preterm (34^0/7^ to 36^6/7^ weeks), early preterm (>28 to < 34 weeks), and very small preterm (≤28 weeks). Our primary outcome was the *mean difference* between axillary and rectal measurements. Sample size calculation was carried for the entire cohort taking into consideration the minimum number of subjects to be recruited from the subgroup of very small preterm newborns while maintaining at least 80% power, since the number of preterm infants born at or below 28 weeks of gestation is small compared to the other gestational age categories. Considering a desirable mean maximum difference between axillary and rectal temperature of <0.3°C, and a mean difference in standard deviation (SD) of <0.5°C (the quoted accuracy of most mercury-in-glass thermometers) [7, 9], the sample size needed to detect a difference of 0.3°C, with SD of 0.5°C, α of 0.05, and power of at least 80% was 24 newborns in each gestational age category.

We used paired t-test to compare the means (SD) of differences between axillary and rectal measurements, and Pearson correlation coefficient (*r*) to investigate the correlation between the two methods. Analysis was done separately for term, late and early preterm newborns. No such analysis was conducted for the very small preterm neonates below 29 weeks of gestation since none met our inclusion criteria during the study conduct. To assess predictors of the difference between the axillary and rectal temperatures, we carried out stepwise multivariate regression analyses, with the outcome being the difference in temperature between the two methods, and the independent variables being those that showed significance at the bivariate association, as well as variables of clinical importance (age, gender, gestational age, birth weight, birth length, mode of delivery, maternal anesthesia, and delivery room resuscitation). To build the model, the entry level of significance was set at 0.1 and the level of retaining variables in the model was set at 0.2.

The degree of agreement between axillary and rectal measurements was assessed using the Bland-Altman plot, which is a scatterplot of the difference of the two measurements against the mean of the two measurements [11]. The plot generates three horizontal reference lines that are superimposed on the scatterplot: one line represents the average difference between the measurements, along with 2 lines that mark the standard deviation of the differences (±2SD). If the two temperature measurement methods are comparable, then differences should be small, with the mean of the differences close to 0, and with no systematic variation with the mean of the two measurements. The Statistical Package for Social Sciences (SPSS, version 21) was used for data management and analyses. A p-value of <0.05 was considered statistically significant.

## Results

During the study period, we enrolled 118 newborns with the following gestational ages: 25 (21%) between 29 and 33 weeks, 30 (25%) between 34 and 36 weeks, and 63 (53%) were equal or above 37 weeks of gestation. Newborns below 29 weeks of gestation could not be recruited because of exclusion criteria. The birth weight ranged between 1,185 and 4,305 grams with a median (IQR) of 2980 (2321.3-3363.8) grams; 60 (50.8%) neonates were males. The cohort’s baseline characteristics are further summarized in Table 1.

**Table 1:** **Baseline characteristics**

Gestational age		29-33 wks	34-36 wks	≥ 37 wks	Total
					29-41 wks
		(N = 25)	(N = 30)	(N = 63)	(N = 118)
**Male gender,** N (%)		12 (48.0)	19 (63.3)	29 (46.0)	60 (50.8)
**Cesarean delivery,** N (%)		21 (84.0)	22 (73.3)	25 (39.7)	68 (57.6)
**Weight-for-date,** N (%)					
Small		1 (4.0)	3 (10.0)	0 (0.0)	4 (3.4)
Large		1 (4.0)	1 (3.3)	9 (14.3)	11 (9.3)
**Nursery setting,** N (%)					
Normal Unit		0 (0.0)	24 (80.0)	62 (98.4)	86 (72.9)
NICU		25 (100.0)	6 (20.0)	1 (1.6)	32 (27.1)
**Maternal anesthesia,** N (%)					
Epidural		2 (8.0)	8 (26.7)	41 (65.1)	51 (43.2)
General		5 (20.0)	2 (6.7)	0 (0.0)	7 (5.9)
Spinal		16 (64.0)	20 (66.7)	15 (23.8)	51 (43.2)
Local/None		2 (8.0)	0 (0.0)	7 (11.1)	9 (7.6)
**Placement,** N (%)					
Crib		10 (40)	25 (83.3)	61 (96.8)	96 (81.4)
Radiant warmer		2 (8)	2 (6.7)	1 (1.6)	5 (4.2)
Closed isolette		13 (52)	3 (10)	1 (1.6)	17 (14.4)
**Resuscitation at birth,** N (%)		7 (28.0)	0 (0.0)	1 (1.6)	8 (6.8)
**Age (Days),** Median		11.0	1.0	1.0	1.0
IQR		6.0-18.0	1.0-1.0	1.0-1.0	1.0-2.3
**Birth weight (Grams),** Median		1765.0	2595.0	3305.0	2980.0
IQR		1482.0-2185.0	2263.8-2761.3	3065.0-3635.0	2321.3-3363.8
**Birth length,** Median		42.0	47.0	50.0	48.0
IQR		40.3-45.0	45.0-48.5	48.5-51.0	45.0-50.0
**Birth head circumference,** Median		30.0	33.0	35.0	33.5
IQR		27.3-32.0	32.9-34.0	34.0-35.5	32.5-35.0
**Crib score,** Range		0-6	0-1	0	0-6
**Axillary temperature (°C),** Mean ± SD		36.8 (0.3)	36.8 (0.3)	36.9 (0.4)	36.8 (0.4)
Range		36-37.4	35.9-37.4	35.8-37.8	35.8-37.8
**Rectal temperature (°C),** Mean ± SD		36.6 (0.4)	36.6 (0.6)	36.9 (0.5)	36.8 (0.5)
Range		35.9-37.3	34.6-37.2	35.5-37.7	34.6-37.7
	**Paired t-test**				
**Comparison of axillary and rectal temperatures**	p value	0.22	0.01	0.55	0.10
	**Pearson Correlation**				
	r (p value)	0.2 (0.30)	0.4 (0.05)	0.6 (0.000)	0.5 (0.000)

The comparison of axillary and rectal temperatures is illustrated in Table 2; the overall mean ± SD axillary (36.8 ± 0.4°C) and rectal (36.8 ± 0.5°C) temperatures were similar, p = 0.1. There was significant overall correlation between both measurements with a Pearson correlation coefficient of 0.5, p = 0.000. When analysis was done separately for each gestational age category, the mean axillary and rectal temperatures were statistically different from each other only in newborns 34–36 weeks gestation (p = 0.01); whereas correlation between the two methods was highest in term newborns (*r* = 0.6, p = 0.000). Interestingly, the strength of the correlation increased steadily with advancing gestational age, from 0.2 at 29–32 weeks to 0.6 at term gestation (Table 1).

**Table 2:** **Linear regression model** ^*^ **for predicting the difference between axillary and rectal temperature**

Predictors	B coefficients	95% CI for B	P value
**Delivery Mode**	0.20	(0.02, 0.38)	0.03
**Chronological age**	-0.01	(-0.02, -0.01)	0.001
**Gestational age**	-0.05	(-0.08, -0.01)	0.008

^*^Variables included in the model were: age, gender, gestational age, birth weight, birth length, mode of delivery, maternal anesthesia, and resuscitation at birth.

**Figure 1:**
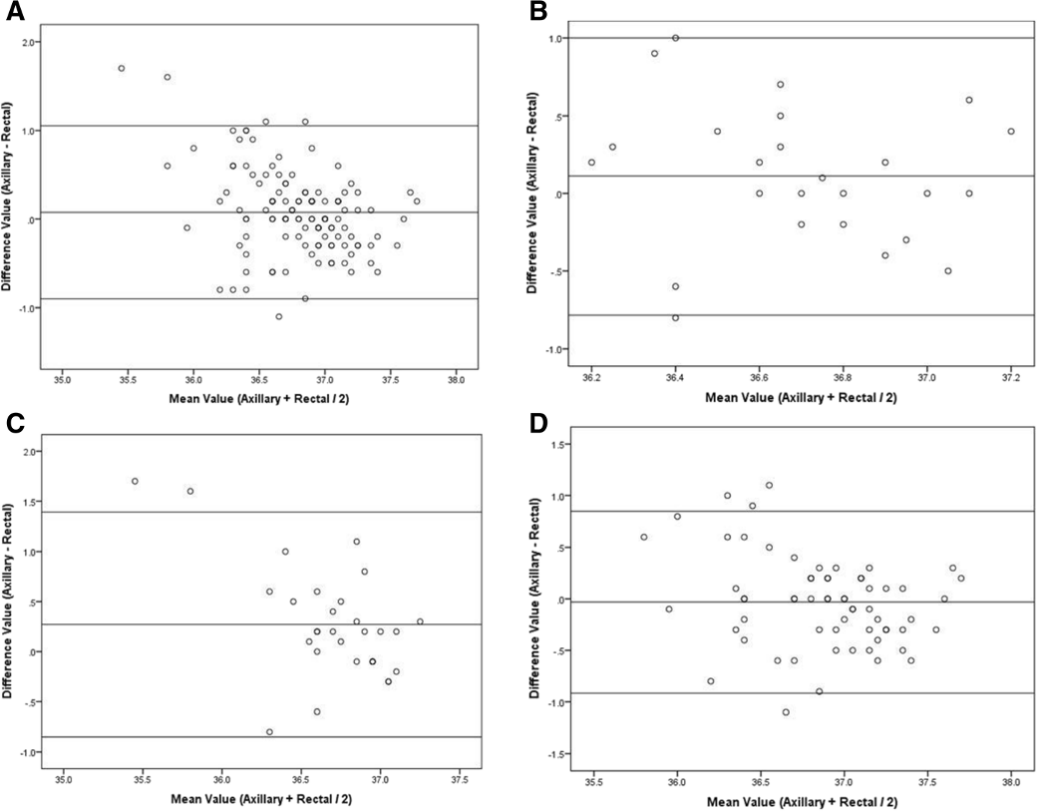
**Bland-Altman plots for the entire cohort (A), 29–33 weeks gestation infants (B), 34–36 weeks gestation infants (C), and ≥ 37 weeks gestation infants (D).**

In the linear regression model, the difference between axillary and rectal temperature was best predicted by the newborn’s delivery mode, chronological age and gestational age (Table 2). Whereas this difference increased significantly with Cesarean delivery (*ß* = 0.2; 95% CI: 0.02, 0.38), p = 0.03, it decreased significantly with increasing chronological age (*ß* = -0.01; 95% CI: -0.02,-0.01), p = 0.001; and with more maturity at birth (*ß* = -0.05; 95% CI: -0.08,-0.01), p = 0.008. The Bland-Altman plots revealed good agreement between the two methods in the overall cohort (Figure 1-A), as well as in each of the gestational age categories (Figure 1B-C, and D), with all the measurements clustering around the zero line difference between the two temperature readings, and within the two standard deviation lines around it.

## Discussion

In this study of clinically stable term and preterm newborns, axillary temperature was in good agreement with rectal temperature measurements. Moreover, there was significant correlation between the two methods, but this correlation was best observed in term newborns. The difference between the axillary and rectal measurements increased with Cesarean delivery but decreased with advancing gestational age and with increasing chronological age.

The main strength of our study is its inclusion of sufficient number of neonates in each of the gestational age categories to allow separate group analysis while still maintaining 80-90% power. Moreover, it is the first study to show that with advancing neonatal maturity and chronological age, there is a higher degree of agreement between axillary and rectal methods, and that Cesarean delivery may reduce this agreement thus decreasing the accuracy of the axillary method. However, since our subjects were clinically stable term and preterm neonates, our findings may not be generalizable to all newborns. Inference to sick neonates or preterm infants born at less than 29 weeks gestation is limited in view of lack of similar infants in our cohort.

Our findings agree with those of Falzon et al. [5] who reported a significant correlation between axillary and rectal temperature (*r* = 0.73, p < 0.0001) but differ with respect to the degree of agreement between the two methods. Whereas we found good agreement, Falzon et al. had poor agreement between the two types of measurements with 95% of axillary measurements falling within 2.5-3°C range around respective paired rectal measurements, using the Bland-Altman method. Additionally, axillary temperatures were consistently lower than rectal ones, with a mean (SD) difference of 0.38(0.76)°C and wide variability. To note, this study included children from birth to 4 years of age but did not provide specific information relating to the subgroup of neonates.

In a larger study that included 282 NICU infants born between 24 and 43 weeks gestation, Helder et al. [8] investigated the correlation between digital rectal and probe skin temperature, measured over the back and the abdomen. Skin temperature measured over the back had a stronger correlation (*r* = 0.77) and better agreement with digital rectal thermometry than abdominal skin temperature (*r* = 0.56). In contrast to our study, Helder et al. found that the correlation between skin and rectal measurements were best for infants with the lowest birth weight (<1000grams; *r* = 0.9; p < 0.001 for back skin temperature) in the first days of life [8], findings that are in an opposite direction to ours. This difference in results may be due to the fact that both studies measured skin temperature using different methods (probe versus digital) and at different sites (back/abdomen versus axilla). In the study of Friedrichs et al. [10], temperature obtained from the left axilla had higher correlation with rectal measurements as compared to that of the right axilla. Our results also contradict those of Hissink et al. [6], Hutton et al. [7], and Lee et al. [9]. Comparing the agreement between axillary and rectal thermometry using the Bland-Altman method, all above studies reported significant differences in healthy and sick term and preterm neonates (range: 25–42 weeks gestation), including those in NICU settings. Moreover, Hissink et al. found that axillary temperature was lower than rectal ones, and that increasing postnatal age increased the difference between the two measurements [6].

## Conclusion

This study provides reassuring evidence regarding the accuracy of axillary thermometry in nursery settings. In newborns at or above 29 weeks gestation that are clinically stable, axillary thermometry is a reliable method for assessing the general well-being of the newborn, therefore guiding decision-making. However, further studies are needed to confirm its accuracy in sick newborns who are clinically unstable and in very small preterm infants less than 29 weeks of gestation.

### Ethical approval

This study was approved by the Institutional Review Board of the American University of Beirut.
